# Supplementary material for: A Surface-Enhanced Raman Spectroscopy-Based Aptasensor for the Detection of Deoxynivalenol and T-2 Mycotoxins
Source: Int J Mol Sci. 2024 Sep 2;25(17):9534. doi: 10.3390/ijms25179534 (PMC11394982; doi:10.3390/ijms25179534)
Supplement: Supplementary file 1 [file ijms-25-09534-s001.zip › ijms-3172211-supplementary.pdf]

A Surface-Enhanced Raman Spectroscopy-Based Aptasensor for the Detection of Deoxynivalenol and T-2 Mycotoxins

Rugiya Alieva [1], Svetlana Sokolova [1], Natalia Zemchuzhina [2], Dmitrii Pankin [3], Anastasia Povolotckaia [3,4], Vasiliy Novikov [4,5], Sergey Kuznetsov [4,5], Anatoly Gulyaev [4], Maksim Moskovskiy [4] and Elena Zavyalova [1,4,*]

1 Chemistry Department of Lomonosov Moscow State University, Moscow 119991, Russia; ruqiwa_eva@mail.ru (R.A.); svetlanasokolova02@mail.ru (S.S.)
2 All-Russian Research Institute of Phytopathology, Bolshiye Vyazemy 143050, Russia; zhemch@mail.ru
3 Center for Optical and Laser Materials Research, St. Petersburg State University, St. Petersburg 199034, Russia; dmitrii.pankin@spbu.ru (D.P.); anastasia.povolotckaia@spbu.ru (A.P.)
4 Federal Scientific Agroengineering Center VIM, Moscow 109428, Russia; vs.novikov@kapella.gpi.ru (V.N.); kuznetsovsm@kapella.gpi.ru (S.K.); tomasss1086@mail.ru (A.G.); maxmoskovsky74@yandex.ru (M.M.)
5 Prokhorov General Physics Institute of the Russian Academy of Sciences, Moscow 119991, Russia
* Correspondence: zlenka2006@gmail.com

Supplementary materials

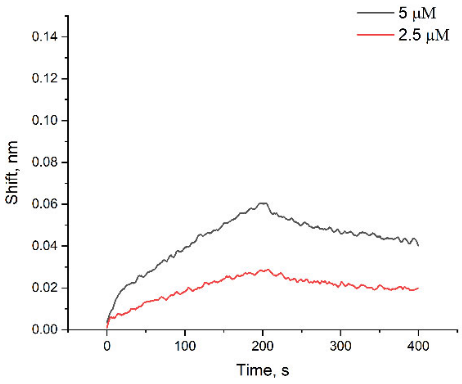

Supplementary Figure S1. Binding curves of Fus aptamer modified with thiol group at the 5’-end.

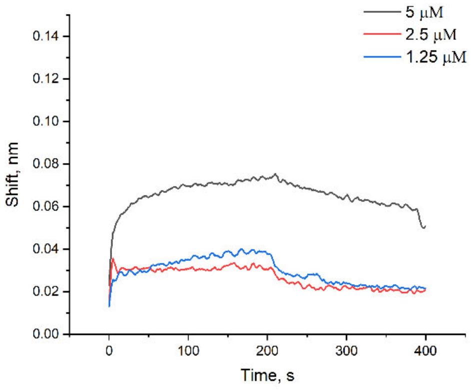

Supplementary Figure S2. Binding curves of Fus aptamer modified with Cyanine-3 (Cy3) at the 3'-end as well as with thiol group at the 5'-end.

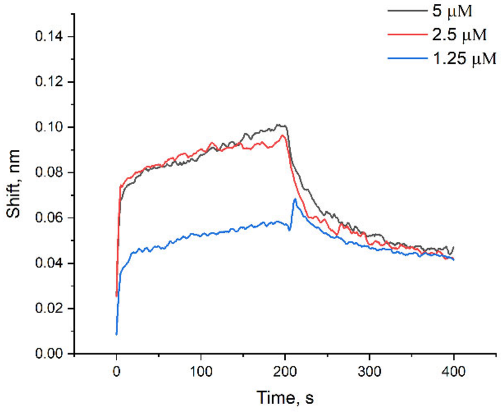

Supplementary Figure S3. Binding curves of Fus aptamer modified with Cyanine-5 (Cy5) at the 3'-end as well as with thiol group at the 5'-end.

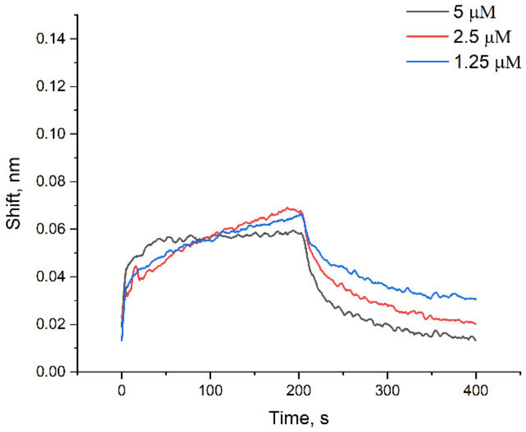

Supplementary Figure S4. Binding curves of Fus aptamer modified with Black Hole Quencher 1 (BHQ1) at the 3'-end as well as with thiol group at the 5'-end.

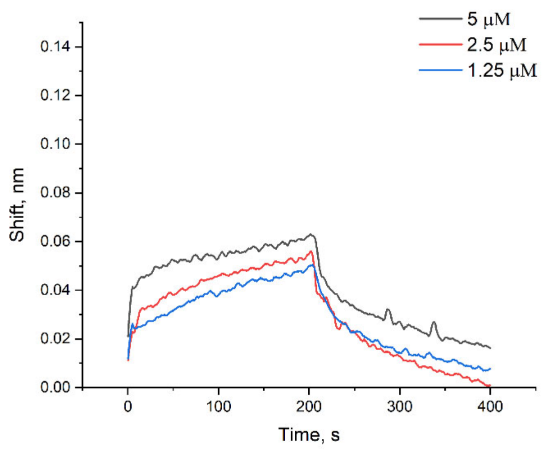

Supplementary Figure S5. Binding curves of Fus aptamer modified with Black Hole Quencher 2 (BHQ2) at the 3'-end as well as with thiol group at the 5'-end.

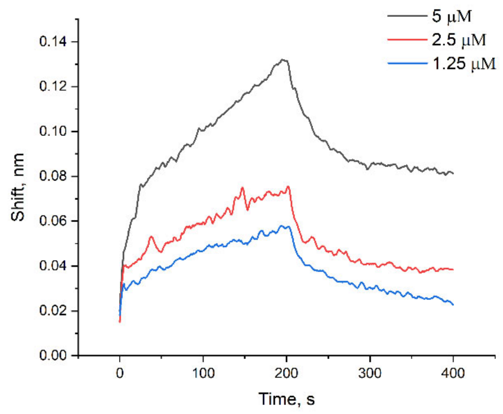

Supplementary Figure S6. Binding curves of Fus aptamer modified with Real Time Quencher 1 (RTQ1) at the 3'-end as well as with thiol group at the 5'-end.

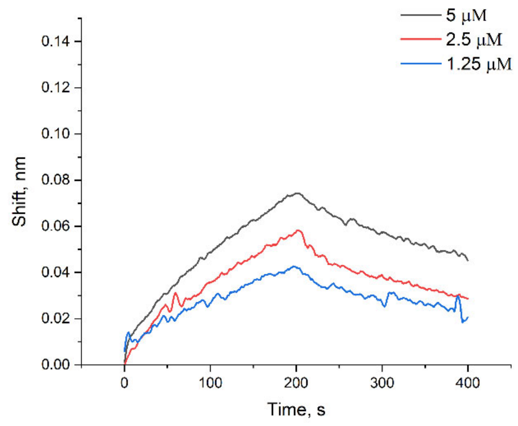

Supplementary Figure S7. Binding curves of Fus aptamer modified with Rhodamine 6G (R6G) at the 3'-end as well as with thiol group at the 5'-end.

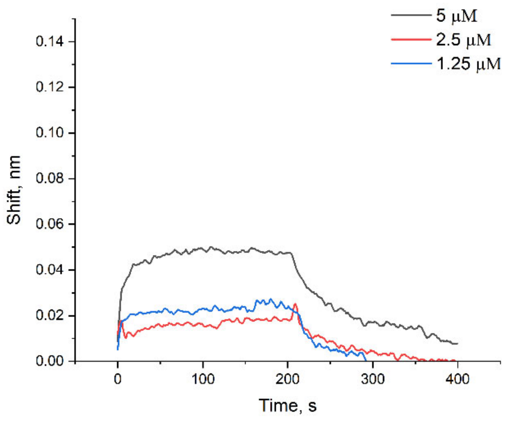

Supplementary Figure S8. Binding curves of Seq16 aptamer modified with Cyanine-3 (Cy3) at the 3'-end as well as with thiol group at the 5'-end.

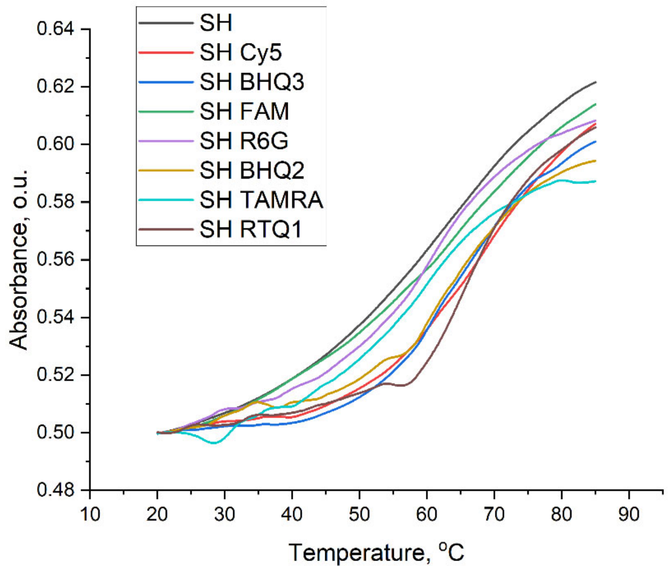

Supplementary Figure S9. UV melting experiment for a series of Fus aptamers with different modifications. The modifications are shown in the capture. The aptamers were assembled in Tris-HCl buffer with pH 7.5, 140 mM NaCl, and 10 mM KCl.

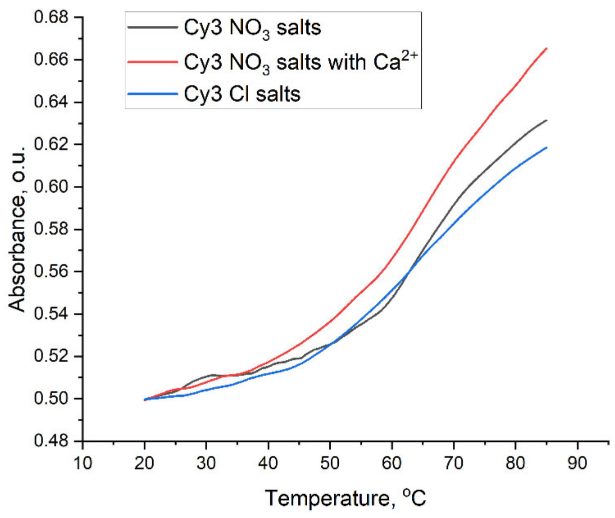

Supplementary Figure S10. UV melting experiment for SH-Fus-Cy3 aptamer in different buffers.

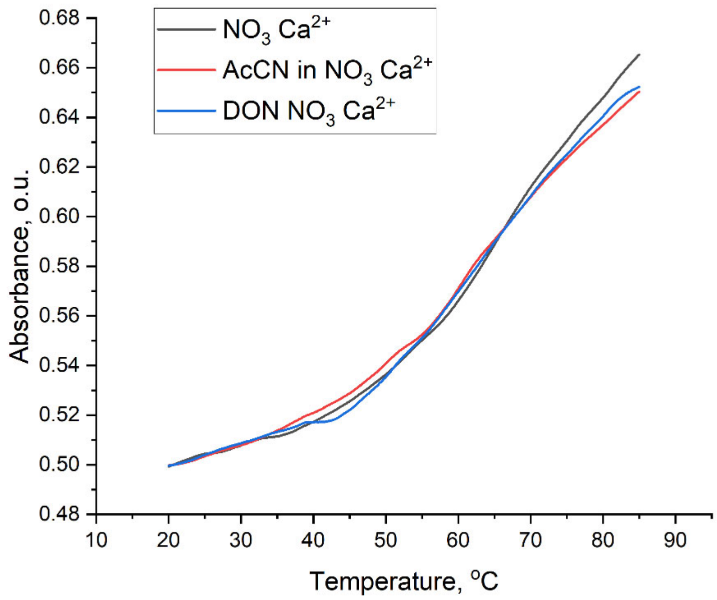

Supplementary Figure S11. UV melting experiment for SH-Fus-Cy3 aptamer in Ca^{2+}-containing buffer in the presence of DON.
